# Winner of The Ronald Melzack – Canadian Journal of Pain 2017 Paper of the Year Award/Récipiendaire du Prix Ronald Melzack Pour L’Annee 2017 Des Articles Parus Dans La Revue Canadienne De La Douleur

**DOI:** 10.1080/24740527.2018.1483111

**Published:** 2018-06-20

**Authors:** Joel Katz

The prize for the Ronald Melzack – Canadian Journal of Pain 2017 Paper of the Year Award was presented to Dr. Gabrielle Pagé at the 39th Annual Scientific  Meeting of the Canadian Pain Society (CPS) in Montreal on May 24, 2018 in recognition of the article titled “Predicting treatment outcomes of pain patients attending tertiary multidisciplinary pain treatment centers: A pain trajectory approach”.^1^ Also in attendance at the ceremony were co-authors, Drs. Manolo Romero Escobar, Mark Ware, and Manon Choinière who share the recognition and honour of the award. Presenting the award were Dr. Laura Stone, CPS Awards Committee Chair and Dr. Gilles Lavigne, *Canadian Journal of Pain* Editorial Board member and Past President of the CPS. The prize included a framed certificate from the CPS and Taylor & Francis, a ticket to the CPS Annual Meeting gala dinner, and a voucher waiving article processing fees for a manuscript to be submitted to the *Canadian Journal of Pain* in 2018.

The winning paper was selected from among 18 original articles published in the *Journal* in 2017 and determined by *Journal* Editorial Board members who were asked to consider originality, novelty, quality, and potential impact when rank-ordering their top three choices. Sixty percent of the voting members ranked the Pagé et al.^1^ article as their number one choice, making it the clear winner.

In a *real-world* study, Dr. Pagé and colleagues examined pre-treatment predictors of improvement and non-improvement among 1,894 chronic pain patients, enrolled in the Quebec Pain Registry, with moderate-to-severe pain intensity and pain interference scores at baseline.^1^ Using growth mixture modeling for multivariate latent classes they examined the patients’ pain intensity and pain interference trajectories over a two-year period using data collected at baseline and at 6, 12, and 24 months. The three-group trajectory solution (non-improvers, steady improvers and early improvers) was collapsed to form a group of improvers (25% of the sample) and non-improvers (75% of the sample) followed by binary logistic regression to predict trajectory group membership from among a variety of pre-treatment risk factors. At baseline, before treatment, improvers (vs. non-improvers) were younger, more likely to have neuropathic pain, had lower pain catastrophizing scores and lower worst pain intensity ratings, fewer depressive symptoms, less disturbed sleep, and higher physical health related quality of life scores. Multivariate general linear modeling at 24 months, showed that in relation to non-improvers, improvers reported fewer depressive symptoms, less disturbed sleep, and higher physical and mental health related quality of life scores than the non-improvers.

These results are sobering. Three-quarters of patients who begin multidisciplinary pain treatment with moderate-to-severe pain intensity and pain interference scores do not improve. Their pain remains relatively constant and severe (between 6/10 and 7/10) across the 24-month period. They are worse than the improvers on a host of other factors as well. Only 25% of patients undergoing multidisciplinary pain treatment show significant improvement in pain severity. The finding, at 24 months, that improvers are also better off than non-improvers on measures of depression, sleep, and quality of life indicates that we are doing something right, albeit, for a select subgroup who are less symptomatic before treatment begins. To the extent that these risk factors for non-improvement are causal, early targeted interventions may help to reverse the pain severity outcomes for some of the 75% of patients who currently do not improve with treatment.

The novelty of the Pagé et al.^1^ study lies in the use of latent class trajectory analysis to identify subgroups of individuals who show a similar pattern of the outcome over time within a class and a different pattern between classes. This differs from more traditional statistical approaches that tend to classify participants using overall averages. As Pagé et al note, pain severity for the entire sample showed a small reduction from baseline to 6 months and little-to-no change thereafter. In contrast, the trajectory analysis permitted a much finer-grained parsing of the data showing three distinct subgroups of patients that differed markedly in pain severity over time.

The *Journal* Editorial Board and the CPS Awards Committee extend their most sincere congratulations to Drs. Pagé, Romero Escobar, Ware, and Choinière for their award-winning article. We look forward to the next competition when we hope to present one award for the basic/preclinical sciences and another for the applied/clinical sciences. Please continue to support the *Journal* with your manuscript submissions. Who knows, you may find yourself at next year’s CPS gala dinner sipping wine and basking in the glory of The Ronald Melzack – Canadian Journal of Pain 2018 Paper of the Year Award/Prix Ronald Melzack Pour L’Annee 2018 Des Articles Parus Dans La Revue Canadienne De La Douleur!
